# Construction of the First Genetic Linkage Map and QTL Analysis for Morphological Traits in *Bougainvillea glabra* Choisy

**DOI:** 10.3390/plants15091373

**Published:** 2026-04-30

**Authors:** Yaru Wang, Ya Li, Shisong Xu, Shengnan Lin, Qingyun Leng, Jinhua Chen, Haiyan Li, Guangsui Yang, Hernán Ariel López, Junhai Niu

**Affiliations:** 1 National Key Laboratory for Tropical Crop Breeding/Key Laboratory of Gene Resources and Germplasm Enhancement in Southern China, Tropical Crops Genetic Resources Institute, Chinese Academy of Tropical Agricultural Sciences, Haikou 571101, China; 2 Hebei Academy of Forestry and Grassland Science, Shijiazhuang 050067, China; 3 Shenzhen Branch, Guangdong Laboratory for Lingnan Modern Agriculture, Agricultural Genomics Institute at Shenzhen, Chinese Academy of Agricultural Sciences, Shenzhen 518000, China; 4 Sanya Research Institute, Chinese Academy of Tropical Agricultural Sciences, Sanya 572024, China; 5 The Engineering Technology Research Center of Tropical Ornamental Plant Germplasm Innovation and Utilization, Danzhou 571700, China; 6 The Botanical Garden of the Plottier City (JBCP), Neuquen 8300, Argentina; hernan_655@hotmail.com

**Keywords:** Bougainvillea, genetic map, QTL mapping, morphological traits, polymorphic markers

## Abstract

Bougainvillea (*Bougainvillea glabra*) is a perennial woody species belonging to the Nyctaginaceae family, native to South America. It is widely recognized for its brightly colored bracts and strong adaptability, and is widely cultivated as a major ornamental plant in tropical and subtropical regions. However, QTL mapping studies on morphological traits remain limited in the genus *Bougainvillea*, and the genetic basis underlying its key ornamental traits is still largely unclear. In this study, an F_1_ segregating population was constructed using the cultivars ‘Mrs Eva White’ and ‘Formosa’ as parents, and was used for high-density genetic map construction and quantitative trait locus analysis. Fourteen leaf and thorn-related traits were investigated, with coefficients of variation ranging from 8.16% to 64.54%. Based on specific-locus amplified fragment sequencing (SLAF-seq), a total of 1,133,206 SLAF markers were developed, among which 479,488 were polymorphic. Using the inclusive composite interval mapping (ICIM) algorithm in the GACD 1.2 software, a high-density genetic linkage map was constructed for B. glabra, consisting of 17 linkage groups and 3256 markers. The genetic map spanned 1797.64 cM, with an average marker interval of 0.55 cM. A total of 22 QTLs were detected for the measured leaf and thorn traits, including 16 major QTLs with a PVE ≥ 8%. Collectively, this study identified stable genetic loci for important morphological traits and provides a valuable theoretical foundation for marker-assisted selection and genetic improvement of ornamental traits in Bougainvillea.

## 1. Introduction

Bougainvillea (*Bougainvillea glabra*) is a popular landscape shrub widely cultivated in tropical and subtropical regions. It possesses high ornamental value due to its brightly colored bracts and foliage. This species exhibits strong adaptability and stress tolerance, making it widely used in landscaping and ecological conservation [1]. Its leaves and bracts are rich in pigments, which can be exploited for the extraction of natural food colorants [2]. Furthermore, various bioactive compounds in bougainvillea, such as flavonoids and phenolic acids, exhibit significant medicinal activities and health-promoting effects [3,4]. As an important ornamental plant, Bougainvillea has attracted extensive attention in genetic breeding research. However, no high-density genetic map covering the whole genome has been constructed to date, which has greatly hindered the progress of QTL mapping and marker-assisted breeding for its key agronomic traits (e.g., flowering time, flower type, and stress resistance) [2,3]. Leaves are vital vegetative organs that provide essential nutrients and energy for plant reproductive growth. Variation in leaf characteristics enhances the ornamental value and visual appeal of bougainvillea. Thorns and pedicels in bougainvillea are homologous organs, and thorn length and morphology are key factors contributing to its phenotypic diversity [3,5]. Genetic analysis of leaf and thorn traits can not only identify valuable genetic loci for trait improvement in bougainvillea.

The main breeding approaches for bougainvillea include cross-breeding, natural bud mutation, and artificial mutagenesis [5,6]. Currently, most new bougainvillea cultivars are derived from bud sports. Bud sports in bougainvillea refers to the spontaneous genetic variation occurring in apical or axillary buds, which leads to phenotypic changes in traits such as thorn morphology, flower color, leaf shape, and growth habit, and serves as an important germplasm resource for the genetic improvement of bougainvillea. Interspecific hybridization can generate abundant genetic variation and thus has great application potential. However, several obstacles, such as small floral organs, low fertility, and strong self-incompatibility, restrict the efficiency of cross-breeding in new variety development [3]. Mutagenesis breeding can significantly shorten the breeding cycle and improve breeding efficiency, and it has also become a major strategy for breeding double-bract cultivars [5].

In recent years, marker-assisted selection (MAS) has emerged as an increasingly important strategy in plant breeding [7,8]. This approach relies on the construction of genetic linkage maps and the development of molecular markers linked to target genes [9]. With the rapid advancement of molecular marker technologies, a large number of markers closely associated with key agronomic and ornamental traits have been identified, thus promoting the wide application of molecular breeding in practical programs. In bougainvillea, the application of SSR markers has greatly facilitated the evaluation of genetic diversity and population structure [10,11]. However, the lack of a high density genetic linkage map has restricted the dissection of the genetic basis underlying important ornamental traits and impeded the progress of molecular breeding in this species.

Specific-locus amplified fragment sequencing (SLAF-seq) allows high-throughput sequencing of target digested fragments throughout the genome. It features high efficiency, flexible restriction enzyme combinations, and no requirement for a reference genome [12]. This technology enables rapid and effective identification of large numbers of SNP and InDel loci, and has been successfully applied to studies on genetic evolution and molecular breeding in ornamental plants without a reference genome [13,14]. For example, SLAF-seq has been used in rose, generating 17,233 high-quality SNP markers from 158 individuals [15]. In eggplant, a high-resolution genetic map containing 2122 SNP markers was constructed using SLAF-seq [16]. In cucumber, a high-density genetic map spanning 845.87 cM with an average marker interval of 0.45 cM was developed from an F_2_ population using SLAF-seq [17].

In this study, SLAF-seq technology was employed to develop SNP markers and construct the first high density genetic linkage map for *Bougainvillea glabra*. QTL mapping was further performed for leaf and thorn morphological traits. The results will provide important genetic resources and a theoretical basis for molecular marker-assisted selection, QTL fine mapping, and candidate gene mining in bougainvillea breeding.

## 2. Materials and Methods

### 2.1. Plant Materials and Growth Conditions

An F_1_ segregating population consisting of 119 individuals was derived from a cross between *Bougainvillea glabra* cv. Mrs Eva White and cv. Formosa. Both parents and the F_1_ progeny were cultivated in the greenhouses of the Tropical Crops Genetic Resources Institute, Chinese Academy of Tropical Agricultural Sciences (TCGRI-CATAS) in Danzhou, Hainan, China (109°50′ E, 19°49′ N).

The plants were maintained under controlled conditions: temperature 18–30 °C and relative humidity 60–80%. They were grown in pots filled with a medium composed of composted coco coir and coarse peat at a volume ratio of 3:1. A fully water-soluble fertilizer with an N:P:K ratio of 20:10:20 was applied every two weeks.

### 2.2. Phenotyping and Statistical Analysis

Phenotypic traits related to leaves and thorns were investigated in the parental lines and 119 F_1_ individuals at the same growth stage over two consecutive years, with at least three biological replicates. Mature leaves from annual shoots were collected to measure leaf length, leaf width, and petiole length using a vernier caliper. Leaf length was measured from the leaf base to the apex, and leaf width was measured at the widest part of the leaf blade. Leaf shape index was calculated from leaf length and leaf width. The leaf shape index was calculated as the ratio of leaf length to leaf width (Leaf length/Leaf width). Lignified thorns at the apex of annual branches were selected for measuring three thorn-related traits: thorn base length, thorn base width, and thorn length. Thorn base length and thorn base width were measured at the base of each thorn, while thorn length was measured from the base to the apex of the thorn. Three biological replicates were measured for each trait, and the average value was used for subsequent analysis.

Excel and SPSS 20.0 software were used for statistical analysis and correlation analysis of leaf and thorn quantitative traits in both parents and the segregating population [18].

### 2.3. DNA Extraction

For DNA extraction, the leaves of the parents and F_1_ offspring were sampled and ground into a powder in liquid nitrogen. DNA extraction was performed using a modified cetyl trimethylammonium bromide (CTAB) method, as described previously [19].

### 2.4. High-Throughput Sequencing and Genotyping

The genomic DNA of the two parents and 119 F1 offspring were used for SLAF library construction and high-throughput sequencing. The genomic DNA was digested to 500–550 bp by RsaI and HaeIII enzymes and sequenced using IlluminaHiSeq platform (Illumina Inc., San Diego, CA, USA).

To improve the accuracy of progeny typing, low-quality SLAF markers with parental sequencing depths of less than 10× and high-frequency variation regions were filtered out. According to the biallelic coding rule, 479,488 polymorphic SLAF tags obtained from the mapping population in this study using SLAF-seq were genotyped. Polymorphic markers of non-aa × bb (homozygous) types were selected as valid markers for genetic map construction, including ab × cc, ab × cd, cc × ab, ef × eg, hk × hk, lm × ll, and nn × np. A further screening was performed on the 267,082 markers eligible for genetic map construction after genotyping. Polymorphic markers were excluded if they met any of the following criteria: parental sequencing depth < 10×, number of SNPs > 5, genotype coverage in the progeny <75%, or segregation distortion (x^2^ test, *p* < 0.0001). After screening, a total of 3752 polymorphic markers were obtained.

### 2.5. High-Density Genetic Map Construction

The 3752 selected SLAF markers were grouped and ordered for genetic linkage analysis and the construction of the genetic linkage map. Two-point linkage analysis was performed to calculate the recombination rate and MLOD value. Markers with MLOD < 5 were filtered out, and the remaining 3256 markers were clustered using the shortest distance method and divided into 17 linkage groups. According to the genotype coding rules of GACD, the markers in the above figure were subjected to genotype replacement. The input files were prepared following the Clonal F_1_ model in the CDM file of GACD 1.2, and the information related to the above markers was imported to construct the genetic linkage map [20]. Collinearity analysis was performed by comparing the positions of bin markers between the physical genome and the genetic map using ALLMAPS software (version 0.8.12) [21].

### 2.6. QTL Mapping

QTL mapping for the 14 quantitative traits was performed using GACD 1.2 software [20]. A permutation test with a 95% confidence interval was conducted to determine the threshold LOD score. The proportion of phenotypic variation explained (PVE) by each QTL was used to classify major QTLs.

## 3. Results

### 3.1. Phenotypic Evaluation of an F1 Genetic Population in Bougainvillea

**Figure 1:**
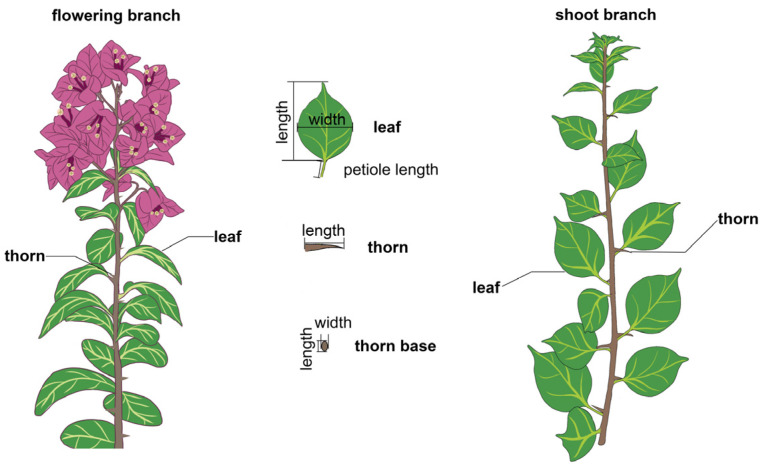
Schematic diagram of the morphology of flowering branch (**left**), shoot branch (**right**), leaf and thorn (**middle**) of bougainvillea.

To identify candidate QTLs for morphological traits in bougainvillea, an F_1_ population comprising 119 individuals was derived from a cross between *Bougainvillea glabra* cv. ‘Mrs Eva White’ and ‘Formosa’ (Figure S1). Leaf and thorn traits differ considerably in size and morphology between flowering branches and shoot branches in bougainvillea (Figure 1). A flowering branch refers to a branch on a bougainvillea plant that has completed the transition from vegetative growth to reproductive growth, differentiated into flower buds, and is capable of directly producing inflorescences. A shoot branch refers to a newly emerged, vigorously growing branch on a bougainvillea plant that sprouts from the base, roots or old branches, focuses on vigorous vegetative growth, has not undergone floral transition, and temporarily has no flowering capacity. To better understand their genetic variation, we investigated and analyzed leaf and thorn traits on both flowering branches and shoot branches. Fourteen quantitative traits associated with flowering shoots and vegetative branches were subjected to statistical analysis to evaluate the genetic variation in leaf and thorn characteristics within the population.

**Figure 2:**
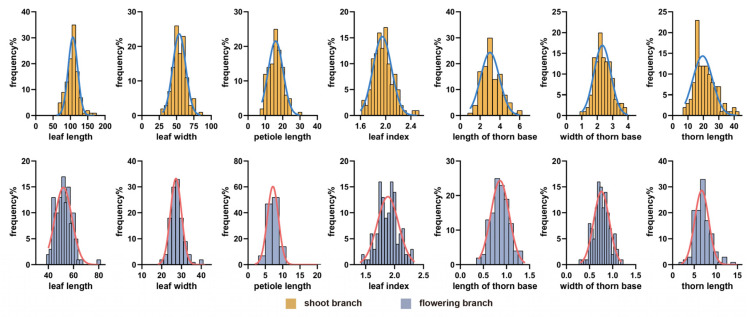
Frequency distribution of morphological traits in the mapping population.

The coefficients of genetic variation (CV) for eight leaf-related traits, including leaf length, leaf width, leaf index, and petiole length, ranged from 8.16% to 27.45% (Table 1). Among these traits, petiole length exhibited the most pronounced variation in the population, with moderate CV values on flowering branches (27.45%) and shoot branches (24.77%). In contrast, the remaining traits (leaf length, leaf width, and leaf index) showed relatively low variation, with CV values below 20%, especially for leaf index. The heterosis values for these traits were predominantly negative, indicating an absence of heterosis for leaf characteristics. The leaf traits displayed a normal distribution in the population, supporting their suitability for QTL mapping analysis (Figure 2).

The coefficient of variation (CV) for six thorn-related traits, including thorn base length, thorn base width, and thorn length, ranged from 22.37% to 64.54% (Table 1). The CV values of these traits on flowering branches ranged from 22.37% to 25.78%, which were significantly lower than those on shoot branches (33.37% to 64.54%). These results indicated that thorn base length, thorn base width, and thorn length exhibited extensive phenotypic variation in the population. Notably, thorn length showed strong heterosis, with heterosis values of 22.15% on shoot branches and 108.31% on flowering branches, suggesting significant heterosis for thorn length. Frequency distribution analysis showed that all thorn-related traits followed a normal distribution, indicating their suitability for QTL mapping analysis (Figure 2).

A comparison of trait performance between the progeny and parental lines revealed that among the eight leaf-related traits, only leaf index showed weak heterosis, while the other traits exhibited no transgressive segregation. Thorn length displayed obvious transgressive inheritance, with heterosis rates of 22.15% on shoot branches and 108.31% on flowering branches. Thorn base length and thorn base width on flowering branches showed similar performance between progeny and parents, whereas these two traits on shoot branches exhibited no transgressive segregation (Table 1).

To clarify the relationships among different phenotypic characteristics, the correlation coefficients among the 14 phenotypic traits in the mapping population were analyzed (Figure S2). The results showed that leaf width and leaf length on shoot branches were significantly positively correlated, with a correlation coefficient of 0.91. By contrast, the correlation coefficient between leaf width and leaf length on flowering branches was 0.67, indicating a moderate correlation. Significant positive correlations were also observed between thorn base length and thorn base width on both shoot branches and flowering branches, with correlation coefficients of 0.85 and 0.95, respectively. No significant correlations were detected between leaf-related traits and thorn-related traits.

### 3.2. SLAF Library Construction and Marker Genotyping

**Figure 3:**
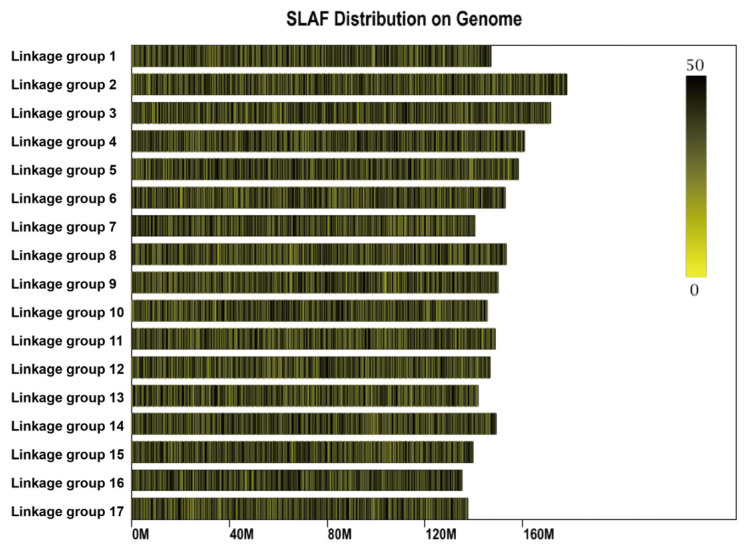
Distribution map of SLAF markers on the linkage maps. The x-axis indicates chromosome length. The color scale bar ranging from 0 (bright yellow, bottom) to 50 (dark olive-brown, top), where color intensity increases with the number of SLAF tags. The higher the number of SLAF tags, the darker the color.

Following SLAF library construction and high-throughput sequencing of genomic DNA from the two parents and 119 F_1_ individuals, a total of 985.72 million raw sequencing reads were generated. The sequencing quality Q30 was 95.43%, and the GC content was 41.86% (Table 2). The distribution plot indicated that the SLAF markers were evenly distributed across all chromosomes (Figure 3). A total of 479,488 polymorphic SLAF markers were encoded following the biallelic coding rule, among which 281,813 markers were successfully genotyped. These markers comprised eight segregation types: aa × bb, ab × cc, ab × cd, cc × ab, ef × eg, hk × hk, lm × ll, and nn × np, with corresponding marker numbers of 14,731, 78,621, 14,132, 18,909, 5357, 297, 11,731, and 5455, accounting for 5.23%, 27.90%, 5.01%, 6.71%, 1.90%, 0.10%, 4.16%, and 1.94% of the successfully genotyped markers, respectively (Table 3). Among these, 14,731 markers belonging to the aa × bb type were homozygous in both parents and therefore not suitable for F_1_ population genetic mapping, representing 5.23% of the successfully genotyped markers. The remaining 267,082 heterozygous markers were used to construct the genetic map, including ab × cc, ab × cd, cc × ab, ef × eg, hk × hk, lm × ll, and nn × np, accounting for 94.77% of the successfully genotyped markers (Table 3). Further screening was conducted on the 267,082 markers that met the criteria for genetic map construction following genotyping. Polymorphic markers were excluded if they met any of the following criteria: parental sequencing depth < 10×, number of SNPs > 5, genotype coverage in the progeny <75%, or segregation distortion (x^2^ test, *p* < 0.0001). After screening, a total of 3752 polymorphic markers were obtained. The 3752 selected markers were then aligned and positioned onto the reference genome and assigned to 17 linkage groups. Markers with an MLOD value < 5 with adjacent markers were removed. Finally, a total of 3256 high-quality SLAF tags were obtained for genetic map construction.

### 3.3. Construction of the High-Density Genetic Linkage Map

**Figure 4:**
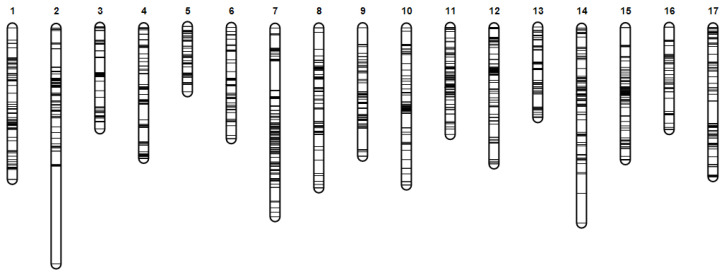
High-density genetic linkage map constructed using GACD.

A total of 3256 high quality markers were selected for genetic map construction after removing markers with an MLOD value < 5. The average sequencing depth was 71.86× in the parental lines and 18.01× in the progeny, respectively. The final genetic map covered a total genetic distance of 1797.64 cM, with an average marker interval of 0.55 cM (Figure 4). LG2 represented the longest linkage group, containing 218 markers and spanning 175.99 cM, whereas LG5 was the shortest, with 138 markers and a genetic length of 53.02 cM (Table 4).

To evaluate the quality of the genetic map, linkage analysis and collinearity analysis were performed. The heatmap revealed strong linkage signals between adjacent markers within each linkage group, and the linkage intensity gradually decreased with increasing genetic distance, supporting the reliability and accuracy of the marker order (Figure S3).

**Figure 5:**
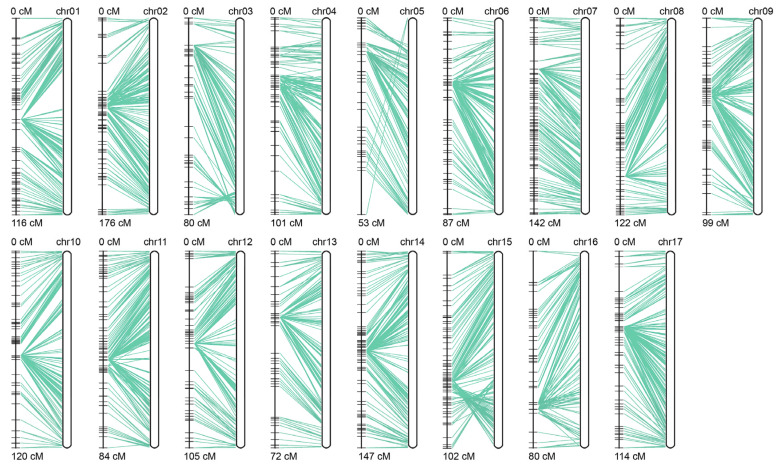
Collinearity analysis between the genetic map and bougainvillea genome sequence.

Collinearity analysis was performed by comparing the positions of bin markers between the physical genome and the genetic map. All bin markers in the genetic map were successfully anchored to the 17 chromosomes of the Bougainvillea reference genome [22] (Figure 5). The order of bin markers within each linkage group was consistent with their physical positions on the genome, indicating high collinearity between the genetic map and the corresponding chromosomes. Nevertheless, a small number of markers of LG3 and LG15 in the genetic map exhibited inconsistent assignments on chromosomes 3 and 15.

### 3.4. QTL Identification for Leaf and Thorn Traits

The QTL mapping of 14 quantitative traits related to leaf and thorn was conducted using GACD1.2 software [20]. The LOD threshold at the 95% confidence level was determined by permutation tests embedded in the GACD 1.2 software. A total of 22 QTLs were identified under the critical condition of LOD ≥ 3.0, explaining phenotypic variation (PVE) from 5.22% to 13.20% (Table S1). A total of 22 QTLs detected by association analysis were distributed across 11 linkage groups. The number of QTLs per linkage group ranged from 1 to 5, and LG9 contained the greatest number of QTLs, with five in total (Table S1).

A total of 11 QTLs were identified for leaf traits, including 8 major QTLs with PVE ≥ 8% (Table 5). Among them, seven QTLs were associated with leaf traits on shoot branches, including six major QTLs: one QTL (QTL-ssll-12) for leaf length, with a PVE of 9.52%, was mapped to LG15; three QTLs for leaf width, with PVE values ranging from 6.47% to 11.18%, including two major QTLs (QTL-sslw-5 and QTL-sslw-7) located on LG6 and LG10; two QTLs (QTL-sslsi-1 and QTL-sslsi-9) for leaf shape index, with PVEs of 12.56% and 9.5%, respectively, both major QTLs on LG1 and LG10; and one QTL (QTL-sslp-6) for petiole length, with a PVE of 9.00%, a major QTL on LG8.

Four QTLs were related to leaf traits on flowering branches, including two major QTLs: two QTLs for leaf shape index (QTL-fblsi-3 and QTL-fblsi-8), with PVEs of 9.1% and 8.67%, respectively, both major QTLs on LG8 and LG12; and two QTLs for petiole length (QTL-fblp-4 and QTL-fblp-7), with PVEs of 5.22% and 5.55%, located on LG9 and LG12.

A total of 11 QTLs were detected for thorn traits, including 8 major QTLs with PVE ≥ 8% (Table 5). Only one major QTL (QTL-sstl-1) for thorn length on shoot branches was identified, with a PVE of 11.76%, located on LG10.

Ten QTLs were associated with thorn traits on flowering branches, including seven major QTLs: one QTL (QTL-fbtl-12) for thorn length, with a PVE of 7.97%, mapped to LG16; four QTLs for thorn base length, with PVE values ranging from 7.67% to 13.20%, including two major QTLs (QTL-fbtbl-6 and QTL-fbtbl-11) on LG7 and LG9; and five QTLs (QTL-fbtbw-3, QTL-fbtbw-6, QTL-fbtbw-8, QTL-fbtbw-9, and QTL-fbtbw-13) for thorn base width, with PVEs from 8.10% to 10.97%, all major QTLs distributed on LG2, LG7, LG9, and LG14.

## 4. Discussion

### 4.1. Genetic Characteristics of Bougainvillea F_1_ Population

Typical quantitative traits are controlled by multiple minor polygenes, and their phenotypic values generally follow a normal distribution. According to the multiple-gene hypothesis, quantitative traits are governed by the independent and combined effects of multiple genes, each with a small individual effect, leading to continuous phenotypic variation [23]. In this study, leaf and thorn-related traits in the mapping population were investigated and analyzed. All 14 traits exhibited segregation in the F_1_ population. The coefficients of genetic variation for the 14 quantitative traits ranged from 8.16% to 64.54%. The degree of variation was higher for thorn traits than for leaf traits, and higher for shoot branches than for flowering branches. Frequency distribution analysis of the 14 traits revealed continuous variation and an approximately normal distribution, confirming that these traits are quantitative in nature and that the mapping population is suitable for QTL analysis.

Heterosis refers to the phenomenon whereby hybrid progeny exhibit superior phenotypic performance compared to their genetically distinct parents [24]. As a crucial strategy in modern crop breeding, heterosis plays an important role in genetic improvement of target traits [25]. In the present study, obvious heterosis was detected for thorn length among the six thorn-related traits, while no over-parent heterosis was observed for the other thorn traits. These results suggest that thorn length presents abundant variation and holds high potential for genetic improvement. Among the eight leaf-related traits, leaf shape index showed weak heterosis, whereas the other leaf traits exhibited no over-parent heterosis. The average leaf length and leaf width of the progeny were lower than the mid-parent values, indicating a declining trend. This pattern is consistent with reports in apple hybrids, where leaf traits also showed a significant downward tendency [26]. Similarly, a low inheritance of leaf length in hybrid progeny has been reported in soybean [27]. Although leaf length and leaf width showed no heterosis, the leaf shape index exceeded that of the parents, suggesting that the genetic inheritance patterns of leaf length and width differed in this progeny population. As a result, progeny leaves tended to be more slender than those of the parents. Collectively, the segregation and genetic characteristics of the 14 phenotypic traits provide a valuable basis for future genetic and breeding research in bougainvillea.

### 4.2. The First High-Density Genetic Linkage Map for QTL Mapping in Bougainvillea

Constructing a genetic map for Bougainvillea can not only elucidate the genetic basis of its complex traits but also provide precise genetic markers for molecular breeding of ornamental traits, filling the research gap in this field. This study demonstrates that the use of an F_1_ population is rational and feasible for dissecting the genetic basis of quantitative traits by applying double pseudo-testcross strategy [28,29]. Intraspecific crosses were conducted between *Bougainvillea glabra* ‘Mrs. Eva White’ and ‘Formosa’, yielding a segregating F_1_ population consisting of 119 progeny individuals. We constructed the first high-density genetic linkage map for bougainvillea, spanning 1797.64 cM with an average marker interval of 0.55 cM. Synteny analysis showed that this genetic map was highly consistent with the ‘Formosa’ reference genome, verifying the accuracy of marker localization and ordering. Further studies will be needed to improve map precision and facilitate QTL fine mapping by integrating multiple genetic maps and increasing marker density in bougainvillea.

Genetic analysis of leaf and thorn traits can not only identify valuable genetic loci for trait improvement in bougainvillea. In this study, a total of 22 QTLs were detected by association analysis based on the constructed high-density genetic linkage map of bougainvillea, combined with phenotypic data for leaf and thorn-related traits. These QTLs were unevenly distributed across 11 linkage groups, with 1 to 5 QTLs per linkage group. Among them, linkage group 9 (LG9) harbored the largest number of QTLs, with five in total (Table S1). This distribution pattern indicates that the genetic loci controlling leaf and thorn traits in bougainvillea are not randomly scattered across the genome, but show obvious clustering. This is consistent with QTL mapping studies in ornamental plants such as tree peony and rose, in which QTLs for key ornamental traits also tend to cluster on specific linkage groups, suggesting that these regions may contain critical genetic hotspots regulating trait expression [29,30].

For leaf-related traits, a total of 11 QTLs were identified in this study, among which 8 were major QTLs with phenotypic variation explanation (PVE) values ≥ 8% (Table 5), reflecting the quantitative genetic characteristic that leaf traits are regulated by both major and minor genes. For thorn traits, 11 QTLs were detected, including 8 major QTLs. Among them, as many as 7 major QTLs were associated with thorn traits on flowering branches, covering thorn length, thorn base length, and thorn base width. These results fully demonstrate that, as the main ornamental and flowering part of bougainvillea, flowering branches have more complex genetic regulation and higher enrichment of major QTLs for thorn traits. This is consistent with QTL studies on prickle traits in rose, indicating that thorn-related traits in woody ornamental plants are mostly controlled by multiple major genes [29]. Overall, major QTLs accounted for 72.7% of the 22 QTLs detected in this study. These findings fill the gap in QTL mapping for leaf and thorn traits in bougainvillea and provide precise genetic loci for subsequent candidate gene cloning, functional verification, and molecular marker-assisted breeding.

## 5. Conclusions

In conclusion, we constructed the first high-density genetic linkage map for *Bougainvillea glabra* and performed QTL mapping for leaf and thorn-related traits. A total of 22 QTLs were detected, including 16 major QTLs significantly associated with leaf length, leaf width, leaf shape index, petiole length, thorn length, thorn base length, and thorn base width. These QTLs were unevenly distributed across multiple linkage groups and exhibited obvious tissue specific expression patterns between vegetative shoots and flowering shoots.

This study not only enriches the genomic resources of bougainvillea but also provides stable and reliable genetic loci for marker-assisted selection breeding. Furthermore, our findings offer a valuable foundation for revealing the genetic basis and molecular regulatory mechanisms of important ornamental traits in bougainvillea.

## Figures and Tables

**Table 1:** Phenotypic statistics of leaf and thorn traits in Bougainvillea parents and F_1_ population.

Traits		Parents			F_1_ Population						
		‘Mrs Eva White’ X ± δ	‘Formosa’ X ± δ	MPs	X ± δ	Max	Min	Skewness	Kurtosis	CV (%)	HI (%)
shoot branch leaf length (mm)	ssll	111.76 ± 20.16	166.45 ± 22.43	139.11	104.73 ± 17.28	155	69	0.17	0.33	16.50	−24.71
shoot branch leaf width (mm)	sslw	59.04 ± 11.74	84.37 ± 7.25	71.71	53.81 ± 9.61	75.35	30.4	−0.04	−0.19	17.86	−24.96
shoot branch leaf shape index (ratio)	sslsi	1.91 ± 0.19	1.97 ± 0.15	1.94	1.96 ± 0.16	2.50	1.63	0.55	0.87	8.16	1.03
shoot branch petiole length (mm)	sslp	17 ± 7.02	26.04 ± 5.25	21.52	15.99 ± 3.96	29	8.25	0.28	0.01	24.77	−25.70
flowering branch leaf length (mm)	fbll	61.64 ± 9.43	61.31 ± 9.10	61.47	51.37 ± 6.25	79	40	0.67	1.78	12.17	−16.43
flowering branch leaf width (mm)	fblw	35.84 ± 5.95	34.98 ± 4.84	35.41	27.42 ± 3.24	40.7	19.35	0.74	2.82	11.82	−22.56
flowering branch leaf shape index (ratio)	fblsi	1.73 ± 0.14	1.77 ± 0.30	1.75	1.89 ± 0.18	2.32	1.43	0.03	−0.13	9.52	8.00
flowering branch petiole length (mm)	fblp	12.43 ± 5.03	6.68 ± 4.84	9.55	7.36 ± 2.02	22.25	3.7	3.35	23.90	27.45	−22.93
shoot branch thorn base length (mm)	sstbl	4.71 ± 0.32	3.76 ± 0.42	4.24	3.13 ± 2.02	5.79	1.2	0.65	0.13	64.54	−26.18
shoot branch thorn base width (mm)	sstbw	2.31 ± 0.14	3.05 ± 0.29	2.68	2.38 ± 0.96	3.85	1.08	0.34	−0.12	40.34	−11.19
shoot branch thorn length (mm)	sstl	18.9 ± 1.10	15.5 ± 2.37	17.2	21.01 ± 7.01	41.25	8.90	0.69	0.24	33.37	22.15
flowering branch thorn base length (mm)	fbtbl	0.80 ± 0.08	0.90 ± 0.12	0.85	0.86 ± 0.20	1.32	0.4	0.08	−0.43	23.26	1.18
flowering branch thorn base width (mm)	fbtbw	0.73 ± 0.09	0.79 ± 0.11	0.76	0.76 ± 0.17	1.2	0.31	0.00	−0.24	22.37	0
flowering branch thorn length (mm)	fbtl	3.13 ± 0.64	3.62 ± 3.04	3.37	7.02 ± 1.81	14.05	2	0.86	2.03	25.78	108.31

MPs indicates mid-parent value, CV indicates coefficient of variation, and Hi indicates heterosis rate.

**Table 2:** Sequencing data statistics of the mapping population.

Sample	Total Reads	Total Bases	Q30 Percentage (%)	GC Percentage (%)
P	35,648,920	7,087,812,734	94.77	42.00
M	42,556,303	8,474,995,968	94.35	41.54
offspring	7,562,659	1,507,222,091	93.87	41.16
Total	985,724,317	196,429,456,630	95.43	41.86
Control	10,374,733	2,073,020,176	94.12	41.68

**Table 3:** Summary of segregation types, parental genotypes, and numbers of SLAF markers in the mapping population.

Marker Numbers	Segregation Type	Parent 1	Parent 2
147,311	aa × bb	a/a	b/b
78,621	ab × cc	a/b	c/c
14,132	ab × cd	a/b	c/d
18,909	cc × ab	c/c	a/b
5357	ef × eg	e/f	e/g
297	hk × hk	h/k	h/k
11,731	lm × ll	l/m	l/l
5455	nn × np	n/n	n/p

**Table 4:** Number and distance of markers on the genetic map.

Linkage Group Group ID	Total Marker	Total Distance (cM)	Average Distance (cM)
LG1	203	115.53	0.57
LG2	218	175.99	0.81
LG3	139	79.95	0.58
LG4	194	100.70	0.52
LG5	138	53.02	0.38
LG6	222	86.87	0.39
LG7	209	142.12	0.68
LG8	212	121.81	0.57
LG9	211	99.05	0.47
LG10	172	119.58	0.7
LG11	229	83.72	0.37
LG12	170	104.68	0.62
LG13	178	71.82	0.4
LG14	216	146.81	0.68
LG15	181	101.80	0.56
LG16	126	80.24	0.64
LG17	238	113.93	0.48
Total	3256	1797.64	0.55

**Table 5:** Major QTLs of leaf-related and thorn-related traits (PVE > 8%).

Phenotypic Trait	QTLs Name	Linkage Group	Position	Left Marker	Right Marker	LOD	PVE (%)
shoot branch leaf length	*QTL-ssll-12*	15	101	Marker969278	Marker337611	3.17	9.52
shoot branch leaf width	*QTL-sslw-5*	6	43	Marker19843237	Marker20092420	4.65	9.07
	*QTL-sslw-7*	10	96	Marker16583718	Marker16797698	3	11.18
shoot branch leaf shape index	*QTL-sslsi-1*	1	65	Marker16285617	Marker15559909	5.92	12.56
	*QTL-sslsi-9*	10	67	Marker16783529	Marker16444421	4.55	9.5
shoot branch petiole length	*QTL-sslp-6*	8	39	Marker6662652	Marker6695139	3.28	9
flowering branch leaf shape index	*QTL-fblsi-3*	8	32	Marker6352472	Marker6594800	3.22	9.1
	*QTL-fblsi-8*	12	12	Marker7388241	Marker7255952	3.1	8.67
shoot branch thorn length	*QTL-sstl-1*	10	20	Marker16622402	Marker16667520	3.7	11.76
flowering branch thorn base length	*QTL-fbtbl-6*	7	138	Marker3518677	Marker2760878	4.06	13.2
	*QTL-fbtbl-11*	9	51	Marker10105828	Marker10285756	3.3	8.55
flowering branch thorn base width	*QTL-fbtbw-3*	2	175	Marker8440685	Marker9294878	3.93	10.02
	*QTL-fbtbw-6*	7	104	Marker3843345	Marker3727030	3.19	8.1
	*QTL-fbtbw-8*	9	25	Marker10603914	Marker10705781	3.71	10.97
	*QTL-fbtbw-9*	9	51	Marker10105828	Marker10285756	4.15	10.38
	*QTL-fbtbw-13*	14	37	Marker22623401	Marker22647615	3.26	8.28

PVE: the phenotypic variation explained. LOD: the logarithm of odds. The LOD threshold for evaluating the statistical significance (*p* < 0.05) of each QTL was set by using a 1000 permutations test.

## Data Availability

The original contributions presented in this study are included in the article/Supplementary Material. Further inquiries can be directed to the corresponding author(s).

## Supplementary Materials

The following supporting information can be downloaded at: https://www.mdpi.com/article/10.3390/plants15091373/s1, Figure S1: Phenotype of the parents in the mapping population: ‘Mrs Eva white’ (left), ‘Formosa’ (right). Figure S2: Correlation analysis and heatmap of 14 morphological traits in the F_1_ population. Figure S3: Evaluation of linkage relationships between adjacent markers on each linkage group. Table S1: List of QTLs detected by inclusive composite interval mapping (LOD ≥ 3.0).
