# Supplementary material for: Construction of the First Genetic Linkage Map and QTL Analysis for Morphological Traits in Bougainvillea glabra Choisy
Source: Plants (Basel). 2026 Apr 30;15(9):1373. doi: 10.3390/plants15091373 (PMC13164872; doi:10.3390/plants15091373)
Supplement: Supplementary file 1 [file plants-15-01373-s001.zip › plants-4156727-supplementary.pdf]

Supplemental Figures

Supplemental Figure S1

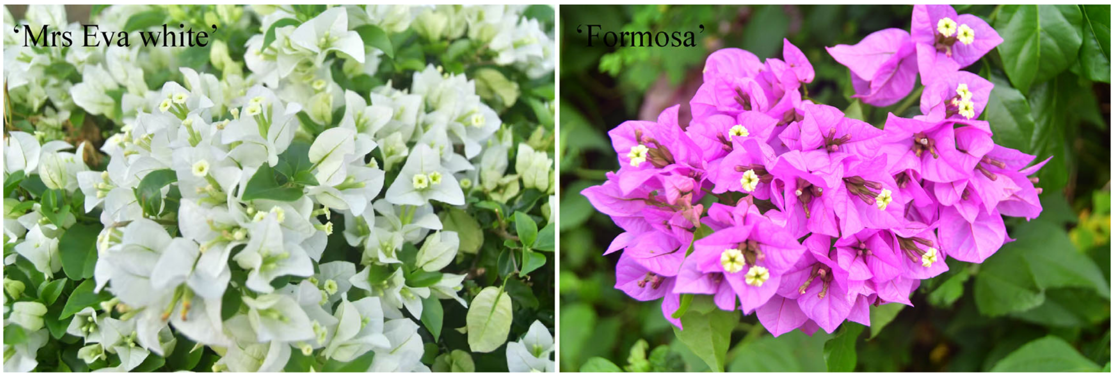

Figure S1 Phenotype of the parents in the mapping population: ‘Mrs Eva white’ (left), ‘Formosa’ (right).

Supplemental Figure S2

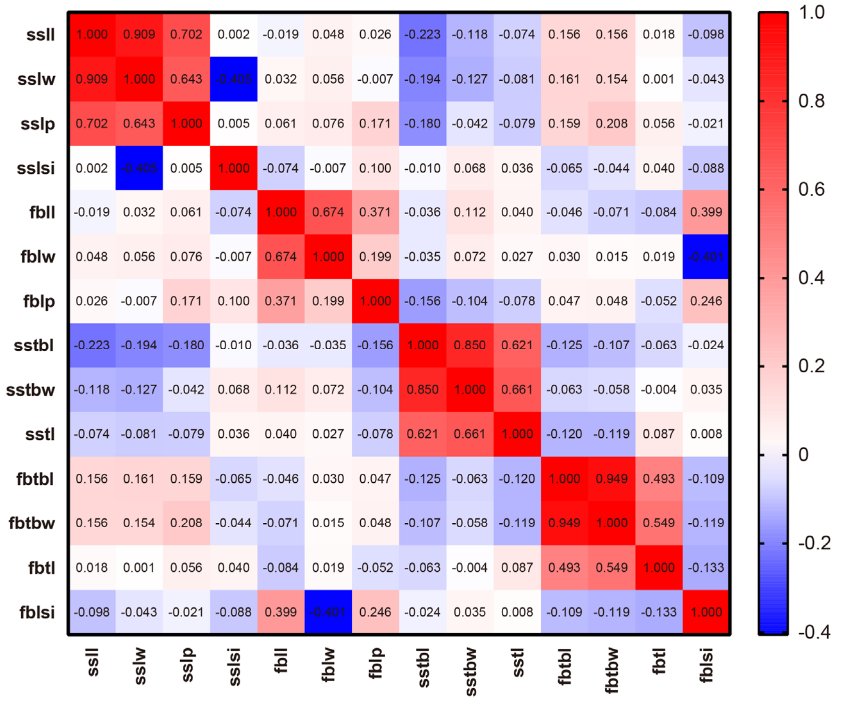

Figure S2 Correlation analysis and heatmap of 14 morphological traits in the F1 population. Color scale bar: A vertical gradient from -0.4 (blue) to 1.0 (red), with white at 0. Values represent correlation coefficients, where color intensity corresponds to the absolute value of the correlation.

Supplemental Figure S3

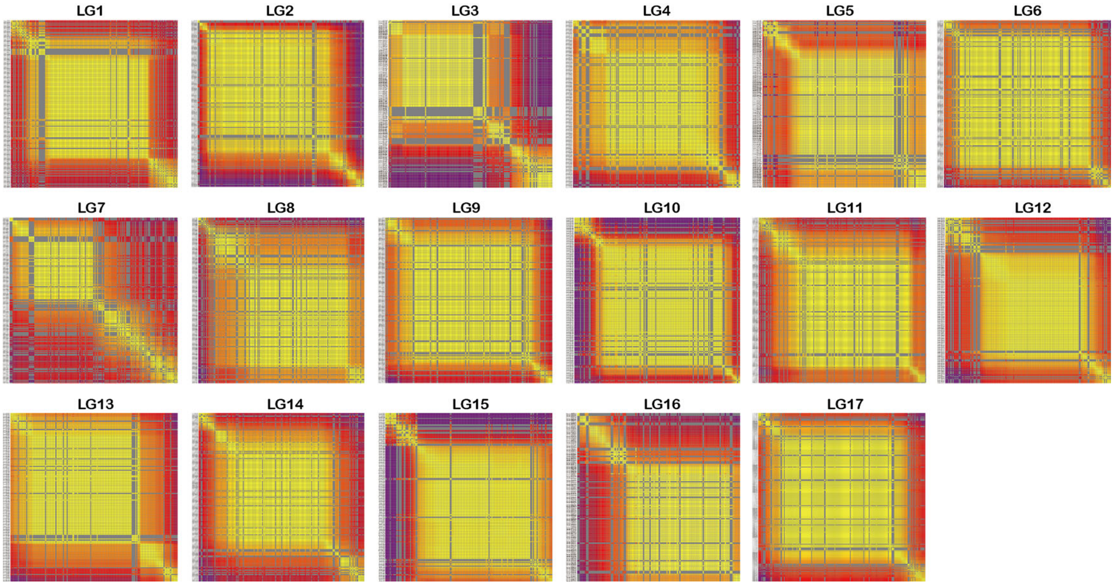

Figure S3 Evaluation of linkage relationships between adjacent markers on each linkage group.

Each row and column represents markers ordered according to the genetic map. Each small square indicates the recombination rate between two markers. The color gradient from yellow to red to purple represents an increase in the recombination rate. Markers that are closer together exhibit a lower recombination rate and appear closer to yellow, whereas markers that are farther apart show a higher recombination rate and appear closer to purple.

Table S1

Table S1 List of QTLs detected by inclusive composite interval mapping(LOD ≥3.0).

Phenotypic trait	QTLs name	Linkage group	Position	Left Marker	Right Marker	LOD	PVE(%)
flowering branch petiole length	*QTL-fblp-4*	9	58	Marker10790700	Marker9778921	3.15	5.22
flowering branch petiole length	*QTL-fblp-7*	12	2	Marker7414526	Marker7479078	3.05	5.25
shoot branch leaf width	*QTL-sslw-6*	9	59	Marker10647630	Marker10877796	3.06	6.47
flowering branch thorn base length	*QTL-fbtbl-13*	16	28	Marker14855961	Marker14279215	3.23	7.67
flowering branch thorn base length	*QTL-fbtbl-8*	8	51	Marker6679011	Marker6643363	3.15	7.72
flowering branch thorn length	*QTL-fbtl-12*	16	28	Marker14855961	Marker14279215	4.27	7.97
flowering branch thorn base width	*QTL-fbtbw-6*	7	104	Marker3843345	Marker3727030	3.19	8.1
flowering branch thorn base width	*QTL-fbtbw-13*	14	37	Marker22623401	Marker22647615	3.26	8.28
flowering branch thorn base length	*QTL-fbtbl-11*	9	51	Marker10105828	Marker10285756	3.3	8.55
flowering branch leaf shape index	*QTL-fblsi-8*	12	12	Marker7388241	Marker7255952	3.1	8.67
shoot branch petiole length	*QTL-sslp-6*	8	39	Marker6662652	Marker6695139	3.28	9
shoot branch leaf width	*QTL-sslw-5*	6	43	Marker19843237	Marker20092420	4.65	9.07
flowering branch leaf shape index	*QTL-fblsi-3*	8	32	Marker6352472	Marker6594800	3.22	9.1
shoot branch leaf shape index	*QTL-sslsi-9*	10	67	Marker16783529	Marker16444421	4.55	9.5
shoot branch leaf length	*QTL-ssll-12*	15	101	Marker969278	Marker337611	3.17	9.52
flowering branch thorn base width	*QTL-fbtbw-3*	2	175	Marker8440685	Marker9294878	3.93	10.02
flowering branch thorn base width	*QTL-fbtbw-9*	9	51	Marker10105828	Marker10285756	4.15	10.38
flowering branch thorn base width	*QTL-fbtbw-8*	9	25	Marker10603914	Marker10705781	3.71	10.97
shoot branch leaf width	*QTL-sslw-7*	10	96	Marker16583718	Marker16797698	3	11.18
shoot branch thorn length	*QTL-sstl-1*	10	20	Marker16622402	Marker16667520	3.7	11.76

shoot branch leaf shape index	*QTL-sslsi-1*	1	65	Marker16285617	Marker15559909	5.92	12.56
flowering branch thorn base length	*QTL-fbtbl-6*	7	138	Marker3518677	Marker2760878	4.06	13.2

PVE: the phenotypic variation explained. LOD: the logarithm of odds. The LOD threshold for evaluating the statistical significance ($p < 0.05$) of each QTL was set by using a 1000 permutations test.
